# The Effects of Intermittent Hypoxic–Hyperoxic Exposures on Lipid Profile and Inflammation in Patients With Metabolic Syndrome

**DOI:** 10.3389/fcvm.2021.700826

**Published:** 2021-08-27

**Authors:** A. Bestavashvili Afina, S. Glazachev Oleg, A. Bestavashvili Alexander, Dhif Ines, Suvorov Alexander Yu, V. Vorontsov Nikita, S. Tuter Denis, G. Gognieva Daria, Yong Zhang, S. Pavlov Chavdar, V. Glushenkov Dmitriy, A. Sirkina Elena, V. Kaloshina Irina, Kopylov Philippe Yu

**Affiliations:** ^1^Department of Cardiology, Functional and Ultrasound Diagnostics, N.V. Sklifosofsky, I. M. Sechenov First Moscow State Medical University, Moscow, Russia; ^2^World-Class Research Center “Digital Biodesign and Personalized Healthcare”, I. M. Sechenov First Moscow State Medical University, Moscow, Russia; ^3^Department of Facultative Therapy, A.I. Nesterov of Medical Faculty, Pirogov Russian National Research Medical University, Moscow, Russia; ^4^Department of Pharmacology (the State-Province Key Laboratories of Biomedicine-Pharmaceutics of China, Key Laboratory of Cardiovascular Research, Ministry of Education), Harbin Medical University, Harbin, China

**Keywords:** metabolic syndrome, inflammation, cholesterol, low density lipopoprotein, high density lipoprotein, intermittent hypoxic training, anti-inflammatory action

## Abstract

**Background:** Patients with metabolic syndrome (MS) tend to suffer from comorbidities, and are often simultaneously affected by obesity, dysglycemia, hypertension, and dyslipidemia. This syndrome can be reversed if it is timely diagnosed and treated with a combination of risk factors-reducing lifestyle changes and a tailored pharmacological plan. Interval hypoxic-hyperoxic training (IHHT) has been shown as an effective program in reducing cardiovascular risk factors in patients with MS even in the absence of exercise. However, the influence of IHHT on the lipid profile and inflammation in this clinical population remains relatively unknown.

**Methods:** A prospective, single-center, randomized controlled trial was conducted on 65 (33 men) patients with MS aged 29–74 years, who were randomly allocated to the IHHT or control (sham) experimental groups. The IHHT group completed a 3-week, 5 days/week intermittent exposure to hypoxia and hyperoxia. The control (sham) group followed the same protocol but was breathing room air instead. The primary endpoints were the lipid profile (concentrations of total cholesterol [TC], low-density lipoprotein [LDL], high-density lipoprotein [HDL], and triglycerides [TG]) and the inflammatory factors such as high-sensitivity C-reactive protein (hs-CRP), galectin-3, heat shock proteins (Hsp70). The secondary endpoints were alanine aminotransferase (ALT), aspartate aminotransferase (AST), N-terminal pro-hormone of brain natriuretic peptide level (NTproBNP), transforming growth factor beta-1 (TGF-beta1), heart-type fatty acid-binding protein (H-FABP), and nitric oxide synthase 2 (NOS2).

**Results:** There were no differences between the two groups but the different baseline values have affected these results. The IHHT group demonstrated pre-post decrease in total cholesterol (*p* = 0.001), LDL (*p* = 0.001), and TG levels (*p* = 0.001). We have also found a decrease in the CRP-hs (*p* = 0.015) and Hsp70 (*p* = 0.006) in IHHT-group after intervention, and a significant decrease in pre-post (delta) differences of NTproBNP (*p* < 0.0001) in the IHHT group compared to the control group. In addition, the patients of the IHHT group showed a statistically significant decrease in pre-post differences of ALT and AST levels in comparison with the control group (*p* = 0.001). No significant IHHT complications or serious adverse events were observed.

**Conclusions:** The IHHT appears to improve lipid profile and anti-inflammatory status. It is a safe, well-tolerated procedure, and could be recommended as an auxiliary treatment in patients suffering from MS, however, the experiment results were limited by the baseline group differences.

**Clinical Trial Registration:**ClinicalTrials.gov, identifier [NCT04791397]. Evaluation of the effect of IHHT on vascular stiffness and elasticity of the liver tissue in patients with MS.

## Introduction

Cardiovascular diseases (CVD) remain the leading cause of morbidity and mortality throughout the world. Risk factors associated with CVD, such as obesity, hypertension, dyslipidemia, and insulin resistance, when combined, define the metabolic syndrome (MS) (1–3). It is estimated that approximately 40–46% of the adult population in the world suffers from MS. The population suffering from MS is at a higher risk of heart attack/stroke, and is three times more likely to have CVD as compared to people without the syndrome (4). In addition, people with MS are at a greater risk of developing insulin resistance and dysglycemia leading to type 2 diabetes (1). At the same time, MS is a reversible condition (2), therefore with an early diagnosis and timely treatment, it is possible to achieve a reduction in the severity of its main clinical manifestations.

There is considerable variability in both the diagnostic criteria and the definition of MS, but in relation to its pathophysiology, the evidence that chronic inflammation underlines MS is growing. On the other hand, its pharmacological treatments are limited by various factors and only partially effective (3).

Besides, the chronic nature of MS typically requires prolonged pharmacological treatment which may not be convenient or affordable for many patients. The development of alternative strategies, such as lifestyle changes, nutraceuticals, education of patients, and other promising novel therapies can limit the side effects and improve patient compliance. In this context, intermittent passive exposure to hypoxia at rest has been identified as a promising strategy, which can reduce cardiometabolic risk factors and become useful in treating patients with exercise difficulties and those who are looking to engage in healthy lifestyles (5, 6). The interval hypoxic training (IHT) has been shown to improve the sympathetic system function, increase the oxygen transport system capacity including capture and utilization of oxygen and energy supply substrates, change lipids and lipoproteins metabolism due to activation of key enzymes that catalyze the esterification of cholesterol and regulate the formation of high-density lipoproteins, decrease the insulin synthesis and insulin response to glucose administration, and reduce renin synthesis so potentially affecting the blood pressure regulation (7–9).

This systemic response may play an important therapeutic role in individuals with insulin resistance, metabolic syndrome, and impaired carbohydrate tolerance (10–12).

A novel approach to IHT, where a “bout” of hypoxia is followed by normoxia, is to alternate hypoxia with mild hyperoxia in order to shorten reoxygenation and stimulate the redox system. The intermittent hypoxic–hyperoxic training (IHHT) program can be viewed as a more effective form of intermittent hypoxia exposure (8, 13). Indeed, during the mild hyperoxia period, a more pronounced induction of reactive oxygen species (ROS) triggers a redox signaling cascade, thus promoting the synthesis of protective intracellular proteins such as antioxidant enzymes and heat shock proteins (14, 15).

The beneficial effects of different passive hypoxic conditioning protocols are well-reported in many studies. The effectiveness of various hypoxic training models has been investigated in the programs of complex treatment and rehabilitation of patients with obesity (16, 17), systemic hypertension (18), and type 2 diabetes (9). The use of hypoxic-hyperoxic training was shown to cause a significant decrease in body weight (mainly due to fat mass reduction) and in blood lipids, lower blood pressure, and improve hypoxia and exercise tolerance. As hypoxic interval training can be personalized, this novel approach has a great potential to play a major role in the complex treatment and rehabilitation of patients with MS by taking individual patient differences into account (6, 19).

The study is aimed to evaluate the effects, safety, and efficacy of the passive systemic IHHT in patients with MS.

## Methods

### Study Design

The study was a single-blind, prospective, randomized (randomization ratio 1:1) parallel-group controlled study performed at the Cardiology Clinic of I.M. Sechenov First Moscow State Medical University, Moscow, Russia.

The study was approved by the ethical committee of I.M. Sechenov First Moscow State Medical University (Local ethics protocol No̱ 05-19 10.04.2019) and carried out in conformity with the ethical standards laid down in the Declaration of Helsinki-Ethical Principles for Medical Research Involving Human Subjects (Bulletin of the WHO [2001]). Written informed consent was obtained from all the trial participants. The study was registered at ClinicalTrials.gov (NCT04791397, protocol ID A0519).

### Participants and Randomization

In the study, 86 patients with MS aged 29–74 years old in stable clinical condition for the last 3 months were invited. The MS was defined according to the National Institute of Health guidelines as having three or more of the following: waist circumference longer than 89 cm in women and longer than 102 cm in men, blood pressure equal to or higher than 130/85 mm Hg, dyslipidemia (triglyceride level 150 mg/dL (1.7 mmol/L) or higher; high-density lipoprotein (HDL) cholesterol <40 mg/dL (1.04 mmol/L) in men, or <50 mg/dL (1.3 mmol/L) in women, elevated fasting blood sugar (100 mg/dL [5.6 mmol/L] or higher). The exclusion criteria were individual intolerance to hypoxia, liver cirrhosis, class C Child-Pugh classification, the patients with positive serological reactions to hepatitis B and C, chronic kidney disease (GFR <30 mL/min/1.73 m^2^), pregnancy, serious respiratory disorders, acute cardiovascular state, and neuromuscular disorders.

**Figure 1:**
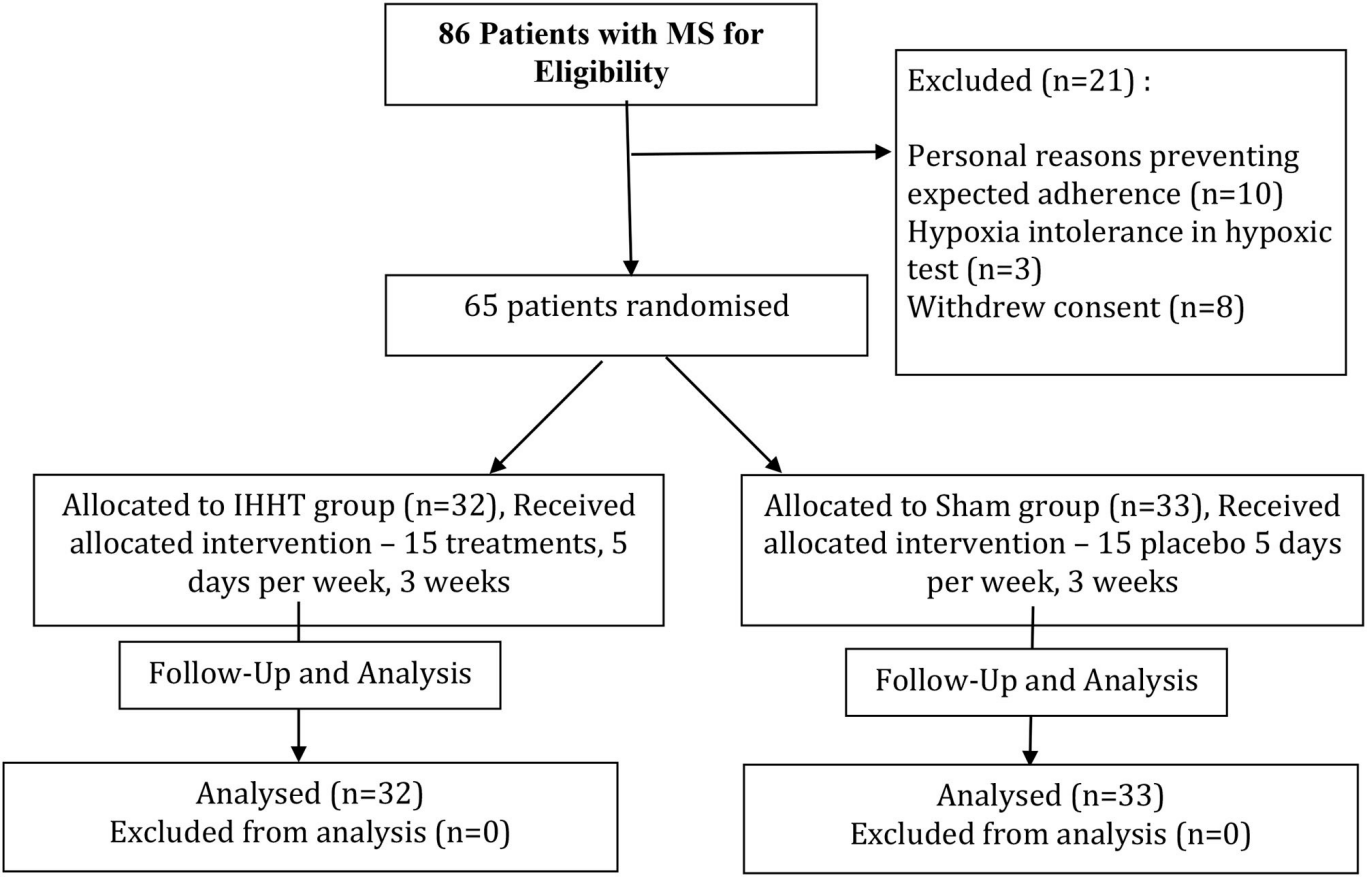
Study flowchart. IHHT, intermittent hypoxic–hyperoxic training; MS, metabolic syndrome.

After the exclusion of 21 patients, 65 patients were randomly (by drawing lots) assigned to either IHHT (32 patients) or Control (sham) (33 patients) group. The process of inclusion, randomization, stratification, hypoxia program, and outcome analysis is presented in Figure 1. The baseline anthropometric, clinical characteristics, and medications are presented in Table 1. The groups were matched by sex, age, presence of MS components, and comorbidities. The patients were asked to maintain their daily food intake, physical activity, prescribed medications, and habitual lifestyle during the whole study period.

**Table 1:** The anthropometric data, morbidity, and medications of the participants.

**Characteristics**	**IHHT group (*n* = 32)**,	**Sham group (*n* = 33)**	***p*-value**
Gender, male	14 (43.7%)	19 (57.5%)	NS
Age, years	60.0 [45.5; 65.5]	61,5 [56.2; 66.0]	NS
Weight, kg	92.0 [81.0; 114]	92.5 [82.8; 104]	NS
BMI, kg/m^2^	34.3 [30.2; 38.0]	32.4 [30.8; 35.8]	NS
Waist Circumference, cm	114 [108; 124]	110 [108; 115]	NS
Hip Circumference, cm	113.5 [106.8; 119.5]	110 [105; 120]	NS
Smoking	10 (31.2%)	11 (33.3%)	NS
Hypertension	32 (100%)	33 (100%)	NS
Diabetes Mellitus, type 2	12 (37.5%)	10 (30.3%)	NS
Obesity (BMI>30)	24 (75.0%)	27 (81.8%)	NS
**Regular Medication:**			
Aspirin	4 (12%)	8 (24.4%)	NS
ACE inhibitors	16 (50%)	13 (39%)	NS
AT II inhibitors	14 (43.7%)	16 (48.4%)	NS
Calcium channel blockers	12 (37.5%)	12 (36.3%)	NS
Beta-blokers	14 (43.7%)	13 (39.9%)	NS
Diuretics	14 (43.7%)	14 (42.2%)	NS
Statins	14 (43.7%)	17 (51.6%)	NS
Metformine	14 (43.7%)	10 (30%)	NS
Sulfonylureas	6 (18.7%)	4 (12.1%)	NS
Insulin	2 (6.25%)	5 (15%)	NS

*Data represent Median (interquartile range 25–75) or frequencies (%)*.

Sixty-five patients have completed the study with their data available for the outcome analysis.

### Intermittent Hypoxic–Hyperoxic Training Program

All patients underwent a hypoxic treatment with the ReOxy Breathing Therapy Device (AI Mediq S.A., Luxembourg). The device delivered a gas mixture of alternating oxygen content (10–35%) and nitrogen. Arterial oxygen saturation (SpO_2_) and pulse rate values were continuously measured using a built-in finger pulse oximeter KIT Masimo (measurement accuracy ± 2%) and stored by the device. After the initial blood pressure measurements, patients from both IHHT and control groups did the same 10 min-long Hypoxic Test (HT) by breathing a hypoxic gas mixture with 11% O_2_ through a face mask while sitting on an armchair. Based on their HT SpO_2_ and pulse rate data, the device automatically calculated and planned individually tailored IHHT sessions for all the subjects (20).

Starting from the next study day, the IHHT group patients have repeatedly inhaled hypoxic gas mixtures with 12–11% O_2_ for 4–7 min (subject to individual hypoxia tolerance as established by the HT), followed by 2–4 min exposure to a hyperoxic gas mixture with 30–35% O_2_. During the sessions, both the SpO_2_ and pulse rate were constantly monitored and transmitted to a monitoring device invisible to the patients. The device compared the latest SpO_2_ value with the predefined individual minimum SpO_2_ level. As soon as a patient reached this minimum SpO_2_, the device immediately switched to the hyperoxic gas mixture until full pre-hypoxic of SpO_2_ level recovery (usually within 1–3 min) occurred; then the next hypoxic–hyperoxic cycle started. Blood pressure was re-measured after the treatment. The overall length of sessions in both the patient groups varied between 40 and 45 min with no observable differences for anyone except the equipment operator/researcher who provided either treatment.

The control group completed a similar program (with the same “exposure” time and number of sessions) but they were breathing a normoxic gas mixture (room air) during the whole session.

In total, 15 hypoxic–hyperoxic or sham treatment sessions were done for both groups five times a week with 2 days weekend break over a period of 3 weeks.

During the first procedures, some IHHT group patients had short-term light dizziness complaints that did not require procedure interruption. After the first procedure, no patients have refused to participate in the further study tests.

### Procedures and Assessments

Before starting the experimental sessions, all the patients underwent a routine medical examination that included checking the daily dosage of medication, family history of hypertension, diabetes mellitus, obesity, and other diseases, by a medical specialist.

In the study, 1-day before starting the course of hypoxic/sham treatments, and 2–3 days after the last treatment session, both groups patients were subjected to the same outcomes measurements: resting blood pressure (BP), heart rate (HR), SpO_2_, anthropometric data (height, body weight, waist, and hip circumference), and blood sampling collection for serum lipid profiles (total cholesterol, triglyceride, direct HDL-cholesterol, and indirect LDL-cholesterol), enzymes (alanine aminotransferase [ALT], units per Liter, aspartate aminotransferase [AST], units per liter), and chronic inflammation markers.

The resting heart rate (HR) and blood pressure values of all the participants were measured twice after 5 min rest in the sitting position using an automatic tonometer AND UA-767 (AND, Japan). The SpO_2_ levels were recorded using a pulse oximeter (Beurer model: PO30, Hollywood, LA, USA). The body weight and height were measured using a beam scale. The waist circumference was measured at the midpoint between the lower margin of the last palpable rib and the top of the iliac crest. The hip circumference was measured at the widest portion of the hip.

#### Venous Blood Samples and Analysis

Samples of venous blood (10 mL) were withdrawn from the forearm median brachial vein and collected into vacuum tubes for the analysis. In order to minimize the platelets count, the blood was allowed to clot (BD Vacutainer Plus SST), and serum was separated immediately (by centrifugation at 3,500 rpm for 15 min after sampling), then aliquoted and stored at −80°C. The samples from a given participant were analyzed in a microtiter plate to minimize the run-to-run variability. All the laboratory analyses were conducted by the University's hospital Blood Analysis Center, a certified blood biochemistry laboratory certified by the Moscow Department of Health.

The levels of brain natriuretic peptide N-terminal prohormone (NTproBNP, N, mediana = 5.6 pmol/L; “Biomedica,” Austria), transforming growth factor beta-1 (TGF-beta1, *N* = 0–2.644 pg/mL; “ThermoFisher Scientific,” Austria), heat shock proteins (Hsp70hs, ng/mL; “Enzo,” New York, NY, USA), galectin-3 (ng/mL; “ThermoFisher Scientific,” Austria), heart-type fatty acid-binding protein (H-FABP, nanograms per milliliter; “Hycult Biotech,” The Netherlands), and nitric oxide synthase 2 (NOS2, nanograms per milliliters; “Cloud-Clone Corp.,” USA; *N* < 0.156 ng/mL) were measured using an enzyme-linked immunosorbent assay and a Biochrom Anthos 2020 Jencons Microplate Reader photometer. High-sensitivity C-reactive protein (CRP-hs, milligram per liter, “Beckman Coulter,” USA, Assay range of 0.2–160 mg/L) was measured using Siemens Advia 1800 Biochemical Analyzer (“Siemens Healthcare Diagnostics Inc.,” USA.) by immunoturbidimetry using latex particles.

The lipid profile and AST/ALT were measured using a Siemens Advia 1800 Biochemical Analyzer (“Siemens Healthcare Diagnostics Inc.,” USA) and dedicated test kits “Siemens Healthcare Diagnostics Inc.,” USA: total cholesterol (TC, ref.: 3.2–5.6 mmol/L), high-density lipoprotein (HDL, ref.: >1.56 mmol/L), low-density lipoprotein (LDL, ref.: 4.2 mmol/L), triglycerides (TG, ref.: 0.4–1.7 mmol/L;), ALT (ref.: 10–49 u/L), and AST (ref.: 0–34 u/L).

### Statistical Methods

All data were analyzed using Python Software Foundation version 3.8 for Windows (Delaware, USA). The data were presented as mean ± SD. The anthropometric characteristics were reported as Median and inter-quartile range 25–75. The assumption of normality and homoscedasticity was verified using Shapiro–Wilk's W-test before parametric tests. To identify the magnitude of statistical difference between Sham and IHHT treatments in each period of the study, Student's *t*-test or Fisher's exact test, as appropriate, was used. In the case of the non-normal distribution of data, we used Mann-Whitney's *U-*test or the Wilcoxon's test to compare the baseline data between the groups as well as different changes (delta pre-post) between the groups. Fisher's exact test was also used to compare the proportions. Pearson's or Spearman's correlation analysis was performed to test relationships between the variables. The α-level was set at 0.05 for all statistical analyses.

## Results

Tables 2, 3 show the descriptive statistics, comparisons, and differences between the two experimental groups for all the measured variables. Due to numerous baseline differences between the groups, the pre-post treatment differences in each group are also shown.

**Table 2:** Anthropometric, lipid profile, and clinically relevant measured variables.

**Variables**	**IHHT group (** ***n*** **=** **32)**		**Sham group (** ***n*** **=** **33)**		**IHHT group, Delta Pre-Post**	**Sham group, Delta Pre-Post**
	**Pre**	**Post**	**Pre**	**Post**		
BMI, kg/m^2^	34.2 ± 5.2	33.3 ± 5.2*p* < 0.001	33.6 ± 4.2	33.8 ± 4.3	−0.9 ± 0.5	0.3 ± 0.6 ^***^p = 0.0001
Waist Circumference, cm	116.2 ± 11.2	111.0 ± 11.6*p* < 0.001	113.1 ± 10.6	113.8 ± 10.9	−5.2 ± 2.4	0.7 ± 1.8 ^***^p = 0.0001
Hip Circumference, cm	114.1 ± 9.4	110.3 ± 9.4*p* < 0.001	112.9 ± 10.6	113.2 ± 11.0 ^**^p = 0.002	−3.8 ± 1.7	3.4 ± 1.0 ^***^p = 0.0001
Total Cholesterol, Mmol/L	6.0 ± 1.3	5.1 ± 1.3	4.8 ± 1.2 ^**^p = 0.001	5.1 ± 1.1 ^*^p = 0.046	−0.8 ± 0.8	0.3 ± 1.0 ^***^p = 0.001
Triglycerides, Mmol/L	2.0 ± 0.6	1.7 ± 0.6	1.6 ± 0.4 ^**^p = 0.01	1.7 ± 0.7	−0.3 ± 0.4	0.1 ± 0.5 ^***^p = 0.001
HDL-Cholesterol, Mmol/L	1.4 ± 0.4	1.4 ± 0.4	1.3 ± 0.6	1.3 ± 0.4	0.0 ± 0.2	0.0 ± 0.2
LDL-Cholesterol,Mmol/L	3.8 ± 1.2	3.0 ± 1.2	2.7 ± 1.1 ^**^p = 0.001	3.0 ± 1.0	−0.8 ± 0.7	0.3 ± 0.8 ^***^p = 0.001
ALT, u/L	37.3 ± 26.1	29.0 ± 15.3^*^p = 0.002	30.8 ± 19.7	36.2 ± 21.5 ^**^p = 0.002	−8.3 ± 14.6	5.4 ± 9.2 ^***^p = 0.001
AST, u/L	31.4 ± 19.0	26.9 ± 10.9	24.9 ± 14.4 ^**^p = 0.006	28.1 ± 16.0	−4.5 ± 12.1	3.2 ± 6.3 ^***^p = 0.001

*Values are expressed as mean ± SD. ALT, alanineaminotransferase; AST, aspartateaminotransferase; HDL-Cholesterol, high-density lipoprotein; LDL-Cholesterol, low-density lipoprotein*.

* *significant difference between Pre and Post in one group*.

** *significant difference between groups at the same study time*.

*** *significant difference between changes in the two groups*.

**Table 3:** Parameters of chronic inflammation.

**Variables**	**IHHT group (** ***n*** **=** **32)**		**Sham group (** ***n*** **=** **33)**		**IHHT group, Delta Pre-Post**	**Sham group, Delta Pre-Post**
	**Pre**	**Post**	**Pre**	**Post**		
Galectin-3, ng/mL	7.39 ± 2.1	7.31 ± 2.31 *p* = 0.08	8.87 ± 3.72	9.49 ± 3.31 ^**^p = 0.003	−0.078 ± 1.641	0.63 ± 1.78 *p* = 0.027
NOS2, ng/mL	0.158 ± 0.154	0.195 ± 0.184	0.07 ± 0.07 ^**^p = 0.001	0.07 ± 0.06 ^**^p = 0.001	0.037 ± 0.104	−0.002 ± 0.011 *p* = 0.317
HSP70, ng/mL	0.963 ± 0.316	0.865 ± 0.334 ^*^p < 0.006	0.818 ± 0.223 ^**^p < 0.03	0.802 ± 0.228	−0.097 ± 0.181	−0.017 ± 0.195 *p* = 0.702
TGF beta1, pg/mL	4,567 ± 7,060	2,609 ± 4,038	7,966 ± 6,860 ^**^p = 0.02	8,412 ± 6,846 ^**^p = 0.001	−1958.119 ± 7045.796	446.206 ± 5803.064 *p* = 0.888
H-FABP, ng/mL	1.52 ± 0.46	1.47 ± 0.54	1.85 ± 0.92	1.96 ± 1.29 ^**^p = 0.02	−0.044 ± 0.269	0.106 ± 0.676 *p* = 0.720
CRP-hs, mg/L	3.608 ± 3.441	2.237 ± 1.527 ^*^p = 0.015	3.51 ± 4.06	3.38 ± 3.49	−1.371 ± 3.534	−0.135 ± 1.803 *p* = 0.904
NTproBNP, pmol/L	27.5 ± 45.1	20.4 ± 34.2 ^*^p < 0.001	25.9 ± 45.9	34.9 ± 62.1 ^**^p < 0.004	−7.1 ± 13.6	9.0 ± 18.0 ^***^p < 0.0001

*CRP-hs, high-sensitivity C-reactive protein; H-FABP, Heart-type fatty acid binding protein; Hsp70, heat shock proteins; NOS2, Nitric oxide synthase 2; NTproBNP, N-terminal pro-hormone of brain natriuretic peptide; TGF-beta1, Transforming growth factor beta-1*.

* *significant difference between Pre and Post in one group*.

** *significant difference between groups at the same study time*.

*** *significant difference between changes in the two groups*.

At the IHHT group baseline, AST (*p* = 0.006), total cholesterol (*p* < 0.001), TG (*p* < 0.015), and LDL (*p* < 0.001) were significantly higher than those measured in the control group. There were no lipid profile differences between groups after the intervention. Nevertheless, analyzing the average difference between the groups pre-post treatment (pre-post delta) demonstrated that the total cholesterol has significantly decreased in the IHHT (TC: −0.8 ± 0.8 mmol/L, *p* = 0.002) group as compared to the control (TC: 0.3 ± 1.0 mmol/L, *p* = 0.001) (*p* < 0.001) group. TG levels were also reduced in the IHHT group in comparison with the control group (*p* < 0.001), and a decrease in the level of LDL in the IHHT group in comparison with the control group (*p* < 0.001) has been observed. The HDL levels were not different between both the experimental groups. In addition, the IHHT group patients exhibited a statistically significant decrease in pre-post differences (deltas) in ALT (*p* < 0.001) and AST (*p* < 0.001) levels as compared with the control group.

The observed post-treatment changes in the inflammatory markers included the decreased levels of CRPhs (*p* < 0.015) and Hsp70 (*p* < 0.006) in the IHHT group, as compared with the control group where there were no post(sham)-treatment differences (Table 3).

The only significant changes in the inflammatory markers in pre-post (delta) intervention between the groups were identified in NT-poBNP (*p* < 0.001) levels in the IHHT group in comparison with the control group.

A tendency toward a decrease in the galectin-3 (*p* = 0.08) levels after the treatment has been detected in the IHHT group, and the levels were significantly lower as compared to the control group (*p* = 0.003). The TGF-beta1 levels were lower both before and after treatment in the IHHT group, and significantly lower after the treatment as compared to the control group (*p* < 0.001). However, due to the variability of values between groups at baseline, no significant pre-post (delta) shifts were found for galectin-3 (*p* = 0.027) and TGF-beta1 (*p* = 0.888).

The statistically significant pre-post changes (delta) in anthropometric indicators were shown: a decrease in the abdominal/hip circumference (−5 cm/−4 cm, *p* = 0.0001) and a decrease in BMI (*p* = 0.0001) in the IHHT group compared with the control group.

## Discussion

We have failed to demonstrate the statistically significant differences between the IHHT and sham patient groups receiving their respective treatments. However, the obtained results were strongly affected by significant baseline differences between both the groups. It is worth noting that the two groups were not different in terms of their anthropometric and clinical profiles, as well as medications taken. At the same time, the baseline values of total cholesterol, triglycerides, and LDL cholesterol were significantly higher in the IHHT group than in the sham group.

Nevertheless, we have also reported statistically significant pre-post changes within each experimental group which could indicate interesting trends in many clinically relevant biomarkers, with specific biomarkers levels improving after 15 sessions in the IHHT but not the sham group.

For instance, measurable decreases in the morphometric indices such as BMI, waist circumference, and hip circumference after 3 weeks of IHHT were not observed in the control group.

Similar reductions in the body weight, adipose tissue mass, and waist circumference have been reported in a number of reviews when different hypoxic conditioning protocols were used (19, 21). The reported body weight reduction promoted by intermittent hypoxic training/exercise is largely due to a decrease in energy intake/appetite suppression accompanied by an increase in energy expenditure and lipid oxidation (6, 22). Nevertheless, most such studies have used active/interval exercising in a hypoxic environment, and the obtained results were inconsistent (23–25).

In contrast to the above studies, we have observed positive effects after using intervallic individually dosed interval passive hypoxic exposure alternating with hyperoxic pauses in the patients with MS without any exercise or dietary changes.

The obesity improvement indicated in the study has been accompanied by a moderate metabolic status correction, namely a decrease in dyslipidemia markers and the liver AST and ALT enzymes. This outcome has been anticipated due to an earlier report by Glazachev et al. (5), wherein uncontrolled study reductions in the total cholesterol, low-density lipoproteins, and glucose after 15 IHT treatments in the patients with MS were reported. The preclinical data and data from healthy volunteers also indicate the potential beneficial effects of moderate hypoxic exposure on blood glucose and cholesterol levels, as well as mitochondrial enzymes activity, glycolysis, and fatty acid oxidation (26–28).

The detected trends in chronic inflammation and cardiovascular health biomarkers are also potentially relevant. Significant intergroup differences in the values of galectin-3 and transforming growth factor-beta1 (TGF-beta1) after the IHHT course were noted. These molecules are considered as profibrotic, proinflammatory, and likely heart and liver fibrosis markers (29). Therefore, their decrease may reflect a reduction in the chronic systemic inflammation typical in the patients with MS (3, 30).

The significant decrease of NTproBNP (*p* < 0.0001) in the IHHT group in contrast to the control group, where the opposite tendency was noted, cannot be ignored. NT-proBNP is a cerebral natriuretic hormone, a protein formed in the left ventricle that plays an important role in the early diagnosis of heart failure. The decreasing value of NT-proBNP can be regarded as a sign of improved cardiovascular function. This is supported by Muangritdech et al. (18) reporting a decrease in blood pressure and improvements of endothelial function/NO availability in the hypertensive patients after a 6-week course of passive hypoxic-normoxic exposures, and further reinforced by data presented in a very recent systemic review confirming antihypertensive IHT/IHHT effects (31).

The pieces of evidence obtained support the idea about the effectiveness of IHHT in the complex treatment of patients diagnosed with MS. Intermittent hypoxia and IHHT could potentially control the development and progression of MS and associated CVD. In animal and human studies, IHC improved the cardiovascular risk factors (CVRF), augmented cerebral blood flow (CBF), and endothelial NO production while decreasing oxidative stress (15).

Intermittent hypoxia-hyperoxia training could be more beneficial than intermittent hypoxia-normoxia (IHT). Indeed, Sazontova et al. (15, 32) has demonstrated in rats that, unlike IHT, IHHT augmented exercise capacity by reducing CVRF, such as arterial hypertension, atherogenic lipid levels, MS, ischemic heart disease, excessive weight, and psychological stress.

In recent decades, IHT has received increased attention as the hormetic approach boosting resistance to common damaging factors (33). Reoxygenation (or in the case of IHHT—mild hyperoxygenation) generate ROS which, if controlled personalized dose (intensity and duration of hypoxic-hyperoxic exposures) is applied, trigger redox-signaling cascades that initiate adaptations to oxidative stress (14, 15). Thus, ROS signaling in the hyperoxic phase facilitates hypoxic stimulus in each treatment bout, so facilitating the conditioning efficiency.

The combination of hypoxic stimuli and hyperoxic pauses in a single procedure has a good physiological foundation in the hypoxic–hyperoxic paradox hypothesis (34). Hypoxia is a natural trigger of mitogenesis and mitochondrial metabolic changes through the induction of hypoxia-inducible factor (HIF), vascular endothelial growth factor (VEGF), other relevant molecular cascades, stem cell proliferation, etc. At the same time, hyperoxic stimuli accompanied by an increased oxygen availability promote the production of both ROS and ROS scavengers, and trigger the same molecular cascades as hypoxia does, activating angiogenesis, mitogenesis, OXPHOS efficiency, and metabolic activity in different tissues.

The safety and efficacy of IHHT shown in the study are corroborated by the successful clinical use of passive hypoxia–hyperoxia exposures in the rehabilitation of elderly patients with dementia and other neurodegenerative diseases (13, 35, 36).

### Limitation

The optimal drug therapy was assessed according to the words from the patients “without checking the drug concentrations in their blood,” which does not exclude increasing adherence to pharmacotherapy in the experimental patient groups. This was a single-centered trial with a relatively small number of patients that does not allow firm conclusions in regard to the rare but important safety events. On the practical side, there may be barriers to the clinical IHHT use due to availability of special equipment and dedicated staff. Our study results only apply to the patients with MS who are clinically stable, so caution should be used in applying the same procedures to other patients.

## Conclusion

This randomized controlled clinical trial has demonstrated that IHHT can be safely used in patients with MS. The observed decrease in the key lipid metabolism and inflammatory markers in the IHHT group supports the efficacy of this intervention in reducing systemic inflammation and improving the lipid profile in patients with MS. Further research is needed to clarify the potential benefits of patient-tailored intermittent hypoxia-hyperoxia programs in clinical populations where inflammation and dyslipidemia play a key pathophysiological role.

## Data Availability Statement

The original contributions generated for this study are included in the article/supplementary material, further inquiries can be directed to the corresponding author.

## Ethics Statement

The studies involving human participants were reviewed and approved by Local ethic committee of I.M. Sechenov First Moscow State Medical University (Sechenov University), protocol No̱ 05-19 10.04.2019. The patients/participants provided their written informed consent to participate in this study.

## Author's Note

The consolidated standards of reporting trial (CONSORT: Consolidated Standards of Reporting Trials) flow diagram is shown in Figure 1 and characteristics of the subjects are presented in Table 1.

## Author Contributions

AAf contributed to the acquisition, analysis, and interpretation of data for the publication, drafting of the publication, writing of the first draft, writing of the manuscript, and the final approval of the manuscript. SO contributed to writing sections of the manuscript, drafting the manuscript, and critically revising it or important intellectual content. AAl contributed to writing sections of the manuscript. GGD agreed to be accountable for all aspects of the manuscript by ensuring that questions related to 858 the accuracy or integrity of any part of the work are appropriately investigated and resolved. SD contributed to writing sections of the manuscript–Methods. SA performed the statistical analysis. DI contributed to the acquisition and analysis of data for the study. VN performed the statistical analysis. VI performed analysis and interpretation of data for the study. YZ substantially contributions to the conception or design of the study. AE and SC contributed to critically revising the important intellectual content. VGD performed analysis and interpretation of data. KP substantially contributions to the conception and design of the study and provided approval for publication of the content. All authors contributed to the manuscript revision and reading and approved the submitted version.

## Conflict of Interest

The authors declare that the research was conducted in the absence of any commercial or financial relationships that could be construed as a potential conflict of interest.

## Publisher's Note

All claims expressed in this article are solely those of the authors and do not necessarily represent those of their affiliated organizations, or those of the publisher, the editors and the reviewers. Any product that may be evaluated in this article, or claim that may be made by its manufacturer, is not guaranteed or endorsed by the publisher.
